# *Salmonella Typhimurium* exploits inflammation to its own advantage in piglets

**DOI:** 10.3389/fmicb.2015.00985

**Published:** 2015-09-22

**Authors:** Barbara Chirullo, Michele Pesciaroli, Rosanna Drumo, Jessica Ruggeri, Elisabetta Razzuoli, Claudia Pistoia, Paola Petrucci, Nicola Martinelli, Lucilla Cucco, Livia Moscati, Massimo Amadori, Chiara F. Magistrali, Giovanni L. Alborali, Paolo Pasquali

**Affiliations:** ^1^Unit of Prophyilaxis and Control of Bacterial Zoonoses, Department of Food Safety and Veterinary Public Health, Istituto Superiore di SanitàRome, Italy; ^2^VISAVET Health Surveillance Centre, Universidad Complutense MadridMadrid, Spain; ^3^Department of Comparative Biomedicine and Food Science, Università degli Studi di PadovaPadova, Italy; ^4^Department of Veterinary Diagnostic, Istituto Zooprofilattico Sperimentale della Lombardia e dell'Emilia RomagnaBrescia, Italy; ^5^S.S. Genova, Istituto Zooprofilattico Sperimentale del Piemonte, Liguria e Valle d'AostaGenoa, Italy; ^6^Research and Development area, Istituto Zooprofilattico Sperimentale dell'Umbria e delle MarchePerugia, Italy

**Keywords:** *Salmonella typhimurium*, inflammation, immune response, pig, salmonellosis

## Abstract

*Salmonella Typhimurium* (*S. Typhimurium*) is responsible for foodborne zoonotic infections that, in humans, induce self-limiting gastroenteritis. The aim of this study was to evaluate whether the wild-type strain *S. Typhimurium* (STM14028) is able to exploit inflammation fostering an active infection. Due to the similarity between human and porcine diseases induced by *S. Typhimurium*, we used piglets as a model for salmonellosis and gastrointestinal research. This study showed that STM14028 is able to efficiently colonize *in vitro* porcine mono-macrophages and intestinal columnar epithelial (IPEC-J2) cells, and that the colonization significantly increases with LPS pre-treatment. This increase was then reversed by inhibiting the LPS stimulation through LPS antagonist, confirming an active role of LPS stimulation in STM14028-intracellular colonization. Moreover, LPS *in vivo* treatment increased cytokines blood level and body temperature at 4 h post infection, which is consistent with an acute inflammatory stimulus, capable to influence the colonization of STM14028 in different organs and tissues. The present study proves for the first time that in acute enteric salmonellosis, *S*. *Typhimurium* exploits inflammation for its benefit in piglets.

## Introduction

*Salmonella enterica* serovar *Typhimurium* (*S*. *Typhimurium*) is a pathogenic Gram-negative bacterium of great clinical significance, responsible for foodborne zoonotic infections. The human disease is characterized by self-limiting gastroenteritis that occasionally can cause fever, systemic infection, and severe inflammation of the intestinal mucosal epithelium (Haagsma et al., 2008; Pires et al., 2011).

The architecture of the mucosal epithelium contains several barriers that prevent or block infection by pathogenic bacteria. Mechanisms of protection are exerted by all of these barriers in order to maintain the integrity of the epithelial cell monolayer and limit inflammation-associated damage (Patel and McCormick, 2014). *S*. *Typhimurium*, however, is able to overcome these barriers and therefore to colonize the intestinal epithelium inducing inflammation and a marked host immune response. The inflammatory response in the gut is induced by the interaction of *S*. *Typhimurium* with host cells including epithelial cells and antigen-presenting cells (APCs), like macrophages and dendritic cells. The inflammation is characterized by the secretion of several cytokines, including interleukin (IL)-23 and IL-18, which in turn stimulates T cells to produce IL-17, and IL-22 in the gut mucosa (Srinivasan et al., 2007; Godinez et al., 2008, 2009; Raffatellu et al., 2008).

*S*. *Typhimurium* acquired an evolutionary adaptation to overcome antimicrobial defenses in the lumen of the inflamed intestine and, more importantly, to exploit inflammation in order to outcompete the intestinal microbiota (Lupp et al., 2007; Stecher et al., 2007; Barman et al., 2008; Lawley et al., 2008; Sassone-Corsi and Raffatellu, 2015). The capability of *S*. *Typhimurium* to grow in the inflamed mucosal environment relies upon the acquisition of essential nutrients and anaerobically respired tetrathionate to successfully outgrow the resident microbiota (Raffatellu et al., 2009; Winter et al., 2010; Liu et al., 2012; Behnsen et al., 2015).

Most of the current studies about *S*. *Typhimurium* infection have been conducted in mice, which naturally do not develop gastroenteritis, but rather a systemic infection. An experimental mouse model using antibiotic treatment in order to eliminate microflora and to induce colitis, has been recently established (Ahmer and Gunn, 2011). However, this model is based on the lack of an intact microbiota, which limits a comprehensive evaluation of the complex interactions of *S*. *Typhimurium* within the gastrointestinal environment (Elfenbein et al., 2013). Here, we utilized pigs as model for gastrointestinal research with the aim of evaluating whether *S*. *Typhimurium* is able to exploit inflammation favoring an active infection. Our findings provide evidence that the LPS administration induces inflammation that favors a significant increase in colonization of tonsils, cecum, and spleen by *S*. *Typhimurium*.

## Materials and methods

### *Salmonella* spp. cultures

A wild-type strain of *Salmonella Typhimurium* ATCC 14028 (STM14028) was used throughout the study. The strain was grown overnight at 37°C in Brain Heart Infusion broth (Oxoid Ltd, UK), harvested by centrifugation at 1500 × g for 10 min and then washed twice in ice-cold (+4°C) phosphate buffer solution (PBS) (Sigma-Aldrich, Italy).

### *In vitro* STM14028 colonization

Porcine peripheral blood mononuclear cells (PBMCs) were isolated from whole blood by Ficoll centrifugation and resuspended in complete RPMI-1640 medium (Sigma-Aldrich, St. Louis, MO) supplemented with 10% fetal bovine serum (FBS, Gibco-BRL, USA), 2 mM L-glutamine, Gentamicin (100 μg/ml). Mono-macrophage cells were isolated from porcine PBMCs, by a 4 h plastic adherence procedure at 37°C in 5% CO_2_ atmosphere, followed by extensive washing with PBS (2 times per day for the first 5 days) to eliminate the lymphocyte contamination. After 7–10 days mono-macrophage cells were obtained, the purity of which was ≥90% as determined by FACS (anti-CD14 Mil-2 mAb AbCAM cat. 23919-1, and anti-pig macrophages mAb, AbD Serotec, cat MCA2317F). Cells were then collected, resuspended in complete medium, and transferred into 200 μL per wells of 96-well round-bottom microtiter plates. The IPEC-J2 cell line, porcine intestinal columnar epithelial cells established from normal jejunum of a neonatal unsuckled pig (ACC 701), were grown in Minimum Essential Medium (MEM) (Sigma-Aldrich, St. Louis, MO) enriched with Fetal Calf Serum (FCS, Gibco-BRL, USA) (10% v/v), 2 mM glutamine, and antibiotics (50μg/mL penicillin, 50μg/mL streptomycin, and 10μg/mL neomycin), at 37°C in 5% CO_2_ atmosphere. Mono-macrophages and IPEC-J2 cell line were employed for *in vitro* studies. Both types of cells were seeded in 96-well plates at a density of 1 × 10^5^ cells per well and treated overnight with purified lipopolysaccharides (LPS) (1 μM/mL; from Escherichia coli 0111:B4, L4391; Sigma-Aldrich) alone or in combination with a natural antagonist of LPS, the RS-LPS (100 μM/mL; tlrl-prslps, Invivogen, San Diego, USA). The following day, cell cultures were rinsed and STM14028 was diluted in RPMI-10% FBS, added to the cells at a multiplicity of infection (MOI) of 100:1 and incubated for 1 h at 37°C in 5% CO_2_. After 1 h, the cell cultures were rinsed and incubated in a culture medium containing gentamicin sulfate (100 μg/ml) to kill extracellular bacteria but not the internal ones, and subsequently incubated for 3 and 24 h. Viable intracellular bacteria were recovered by lysing the cells, at both 3 and 24 h post treatment time point, in distilled water with 0.1% of Triton X-100 for 10 min. The quantification of bacteria was performed by plating serial dilutions on agar triptose plates.

### *In vivo* studies

#### Animals

Fourteen commercial hybrid pigs aged ~30 days were utilized in the experiment. All the pigs used throughout the study were the offspring of *Salmonella*-free sows (negative for *Salmonella* by both serological and bacteriological tests). Before the onset of the experiment, the piglets were proved to be *Salmonella*-free by culture of feces of each animal. Animals were weighed and randomly allocated to two groups of 6 (A and B) and one group (C) of two pigs. Each group was maintained in separate isolation units under natural day–night rhythm with access to feed and water *ad libitum*. Groups A and B were intragastrically administered with 20 ml of sodium bicarbonate buffer containing 10^9^ CFU of *S*. *Typhimurium* ATCC 14028. At the same time, Group A was intraperitoneally challenged with 12.5 μg/kg BW of lipopolysaccharides from *S. enterica* serovar *Typhimurium* (L2262, Sigma Aldrich SRL, Milan, Italy). Group C received only sterile sodium bicarbonate buffer and served as naïve control group. Collection of individual fecal samples (0, 1 day after challenge), blood sampling and registration of rectal temperature (0, 4 h, 1 and 2 days after challenge) were performed as well. Pigs were visually monitored by an independent veterinary officer in charge of the study for 6 h after the inoculum and then twice a day. Two days after the challenge, pigs were weighed again, and then euthanized using a captive bolt pistol and exsanguination. Samples of tonsils, liver, spleen, mesenteric lymph nodes, ileum, cecum, and colon were collected from each pig for the evaluation of bacterial burden.

All the experiments were authorized by national authority and were conducted according to the Italian national regulations enforced at the time of this study (Italian legislative Decree 116/92).

#### Microbiology

The microbiological analysis of fecal and organ samples were conducted according to the ISO 6579:2002/Amendment 1:2007 protocol. Briefly, samples were weighed and homogenized as 10% suspension in Buffered Peptone Water (BPW) (Oxoid Ltd., UK). This initial solution was then used to perform a decimal dilution series carried out by systematically transferring an aliquot of 1 ml of each successive dilution in 9 ml of BPW. All BPW-diluted samples were incubated at 37°C for 18 ± 3 h. Cultures (0.1 ml) were subsequently seeded on Modified Semisolid Rappaport-Vassiliadis (MSRV) agar plates (Oxoid Ltd., UK) and incubated at 41.5°C for 24 h for the selective enrichment of *Salmonella*. A loopful of growth from each MSRV plate was streaked onto Xylose-Lysine-Desoxycholate Agar (Oxoid Ltd., UK) and Brilliant Green Agar (Oxoid Ltd., UK) plates and hence incubated at 37°C overnight. Typical colonies were confirmed serologically as *Salmonella* by polyvalent antiserum (*Salmonella* Test Serum; Siemens Healthcare Diagnostics, Italy) and API rapid 20 E (Api Rapid 20E; Biomerieux, Italy). This is a semi-quantitative approach that allow the determination of the concentration of *Salmonella* in a sample within a tenfold band.

#### Flow cytometry of lymph nodes cells

Cell treatment was performed according to an established procedure (Razzuoli et al., 2012), with minor modifications. Briefly, frozen lymph nodes cells were thawed at 38°C and washed with FACS-Buffer (0.1% sodium azide + 2% fetal calf serum in PBS). Then, they were divided into aliquots (10^6^ cells each) and reacted with monoclonal antibody (mAb) CD21 (Southern Biotech, cat. 4530-02), Mil-2 (AbCAM, cat. 23919-1), PMN (AbD Serotec, cat. MCA2599F), or FACS buffer only (control) for 30 min at 4°C, respectively. Cells were washed, and again incubated for 30 min at 4°C in FACS buffer containing goat anti-mouse IgG-FITC (Invitrogen, Molecular Probes®, cat: A10683). After washing in FACS buffer, cells were resuspended in 100 μL of the same buffer and 1:4 diluted. Samples were analyzed in a GUAVA MILLIPORE flow cytometer (Millipore Software). The typical forward and side scatter gate was set to exclude dead cells from the analysis. The percentage of positive cells beyond the threshold fluorescence channel was assessed in each sample on 10,000 events and compared between mAb-treated and control cells. For each antibody, results were expressed in terms of net percentage of positive cells.

#### Analysis of cytokines

In order to evaluate the serum concentration of IL-1 beta and TNF-alpha ELISA kits were used (cat. N. PLB00B and PTA00 respectively, R&D Systems, Inc. Minneapolis, MN 55413, USA,). These assays employ the quantitative sandwich enzyme immunoassay technique using monoclonal antibodies, specific for porcine IL-1 beta or porcine TNF- alpha, pre-coated onto a microplate. The intensity of the color measured is at 450 nm.

#### Statistical analysis

For the *in vitro* and *in vivo* assays, the statistical significance of differences between study groups was analyzed using ANOVA and Student's *t*-test; *p* < 0.05 was chosen as threshold for significance. These symbols were used to indicate the statistical significance: ^*^P < 0.1; ^**^P < 0.01; ^***^P < 0.001; ^****^P < 0.0001.

## Results

### *Salmonella* infection induces an innate immune response

**Figure 1:**
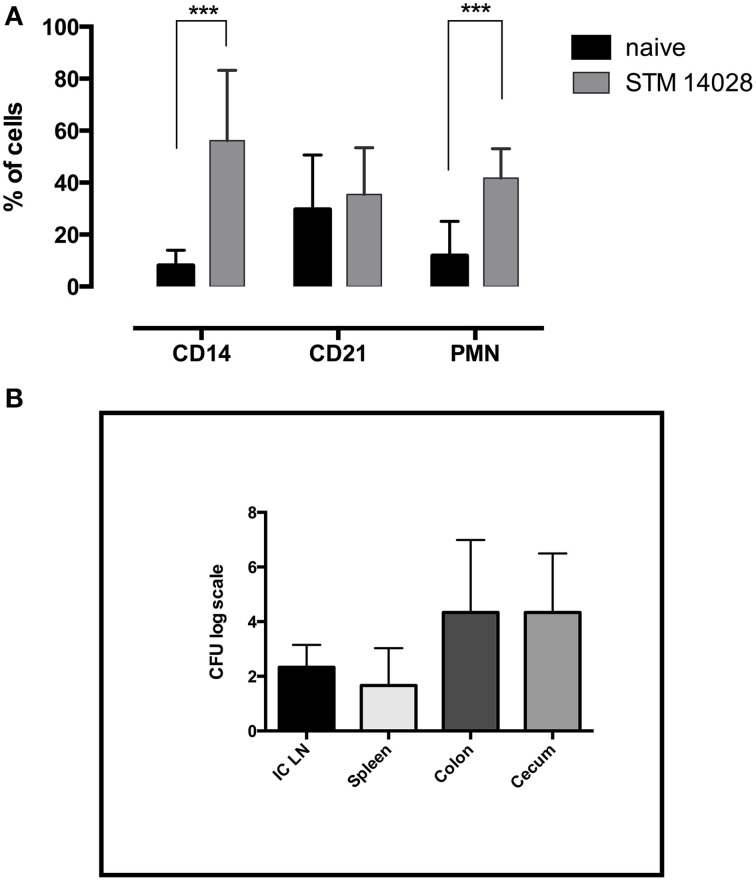
**STM14028 infection induces an increase of innate immunity cell compartment and colonizes different organs of piglets orally infected**. **(A)** The prevalence of CD14^+^, CD21^+^, and polymorphonuclear (PMN) cells was determined in ileo-cecal lymph nodes, 48 h post-infection with STM14028. The differences were statistically significant (^***^P ≤ 0.001, multiple comparisons *t*-test). **(B)** STM14028 count in ileo-cecal lymph nodes (ICLN), spleen, colon, and cecum of infected piglets. Data represent mean with error bars as SEM of six piglets per group.

In order to verify the involvement of the innate immune response during *Salmonella* infection in piglets, phenotypic analysis in ileo-cecal lymph nodes was performed in piglets orally infected with STM14028 and euthanized 48 h later. As depicted in Figure 1A, it was possible to observe a higher increase in the percentage of CD14^+^ (mainly monocytes) and polymorphonuclear cells with a minor involvement of CD21^+^ B cells. Moreover, the STM14028-infection was confirmed by the bacterial count in different organs (Figure 1B). These findings are consistent with a rapid recruitment of neutrophils and monocytes/macrophages toward lymph nodes, crucial for the effective response to lipopolysaccharides stimulus or concomitant bacterial infection. These data suggest a prompt involvement of the innate immune response in the regional lymph nodes draining the gut, after an oral infection with STM14028.

### LPS treatment increases STM14028 colonization in isolated mono-macrophages and IPEC-J2 cells

In order to assess if STM14028 exploits inflammation *in vitro*, we used LPS, which is known to stimulate the production of inflammatory molecules either in monocytes/macrophages (Fang et al., 2004) or in IPEC-J2 cells (Razzuoli et al., 2013). Monocytes/macrophages and IPEC-J2 cells were thus primed overnight with purified LPS alone and/or in combination with a natural antagonist of LPS (RS-LPS) capable to inhibit the LPS-stimulation interacting with the TLR-4/MD-2 complex recognized by LPS. Afterwards, the cells were infected with STM14028.

**Figure 2:**
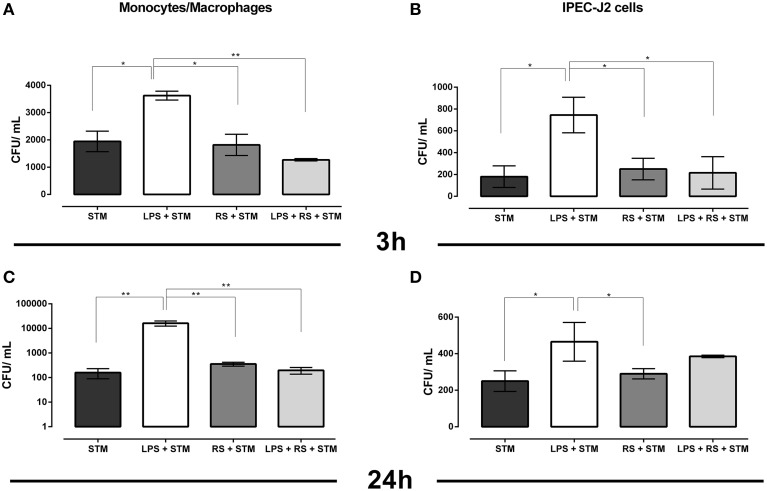
**STM14028 colonization of mono-macrophages and IPEC-J2 cells at 3 and 24 h post infection (A–D)**. STM14028 colonization increases with LPS pre-treatment and is reduced by RS-LPS antagonist to the values of LPS-untreated cells (^*^P ≤ 0.1; ^**^P ≤ 0.01, One-Way Anova Turkey's multiple comparisons test, data from one representative experiment out of three with similar results).

We observed that STM14028 was able to efficiently colonize monocytes/macrophages and IPEC-J2 cells at both 3 and 24 h post STM14028-infection (Figures 2A–D). A previous treatment with purified LPS significantly increased STM14028 colonization in both cell types at 3 and 24 h after infection. When RS-LPS antagonist was used, this markedly inhibited LPS stimulation causing a colonization level similar to the one obtained by STM14028 infection alone (Figures 2A–D). Overall, these results suggest that LPS stimulation can create conditions in which STM14028 is more efficiently phagocytized by cultivated cells, within it can find suitable conditions to multiply.

### LPS induces inflammation in piglets, which in turn favors STM14028 colonization

To evaluate the influence of inflammation during *Salmonella* infection *in vivo*, we established a protocol of inflammation, injecting parenterally LPS in group A and STM14028 by oral route in groups A and B. Piglets in group C were kept untreated and served as control animals. Then, an assessment was made of whether inflammation induced by LPS had favored the progression of *Salmonella* infection.

**Figure 3:**
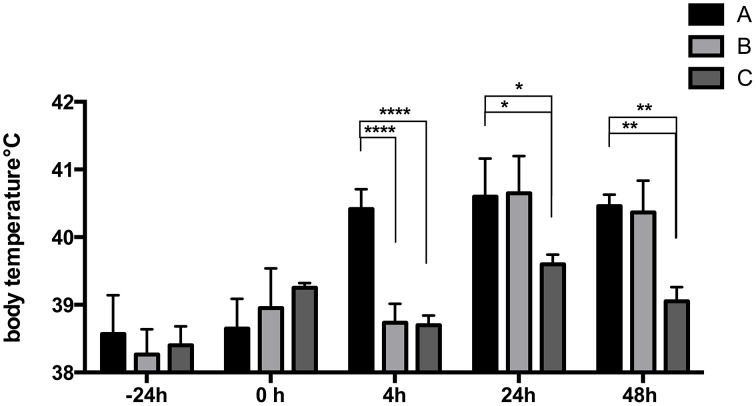
**LPS-treatment of piglets induces a rise in body temperature 4 h after infection with STM14028**. The body temperature was measured at different time points on three different groups of piglets: treated with LPS and infected with STM14028 (group A); only STM14028 infected (group B); naïve control group (group C). At 4 h post infection, group A showed a significant rise in body temperature compared to the B and C groups. Data refer to one out of two separate experiments performed with comparable results. The differences between groups were statistically significant (^****^P ≤ 0.0001; ^*^P ≤ 0.1; ^**^P ≤ 0.01 multiple comparisons-Fisher's Least Significant Difference test).

LPS was able to induce a rise in body temperature in piglets of group A already at 4 h post STM14028-infection (Figure 3) compared to the control (C) and the STM14028-infected group (B), reaching body temperature similar to those of group A only at 24 and 48 h post infection. No significant differences in body weight were measured among the three groups throughout the 48 h of analysis (data not shown).

**Figure 4:**
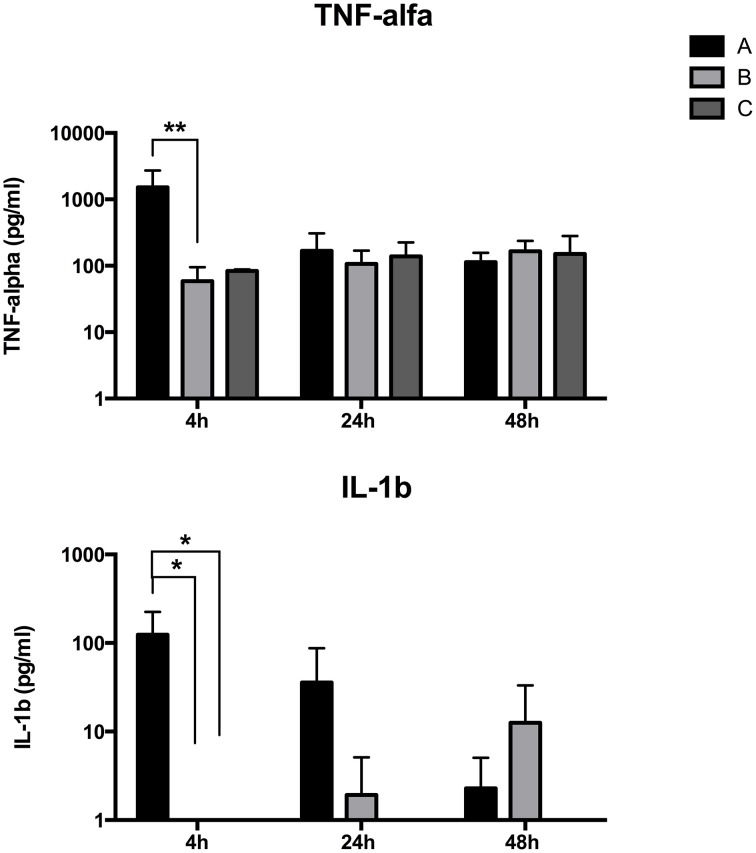
**LPS-treated piglets show an increased inflammation 4 h post STM14028 infection**. IL-1beta and TNF-alpha production was measured at different time points on blood samples from three different groups of piglets: treated with LPS and infected with STM14028 (group A); only STM14028 infected (group B); naïve control group (group C). At 4 h post infection, group A showed a significant increase in production of both cytokines compared to the B and C groups. The differences between the groups were statistically significant (^*^P ≤ 0.1; ^**^P ≤ 0.01, multiple comparisons-Fisher's Least Significant Difference test).

Moreover, a remarkable increase of circulating pro-inflammatory cytokines has been found (Figure 4). TNF-alpha was in fact detected in the blood of all animals but it was produced in a higher amount in group A compared to the groups B and C, at 4 h post STM14028-infection, without significant differences within groups at 24 and 48 h post infection.

IL-1 beta blood level was higher in group A than in groups B and C at 4 h post STM14028-infection. At 24 h post infection we detected a reduction of the IL-1 beta blood concentration in group A with a concomitant slightly increased level in group B. Finally, the IL-1 beta level completely reversed its trend in groups A and B at 48 h post infection, with higher concentration of IL1-b in group B compared to group A (Figure 4).

These results confirm the induction of a pro-inflammatory status mediated by LPS immediately after its administration. Noteworthy, in group B the level of pro-inflammatory cytokines required 48 h after infection to reach a concentration similar to that measured in group A.

**Figure 5:**
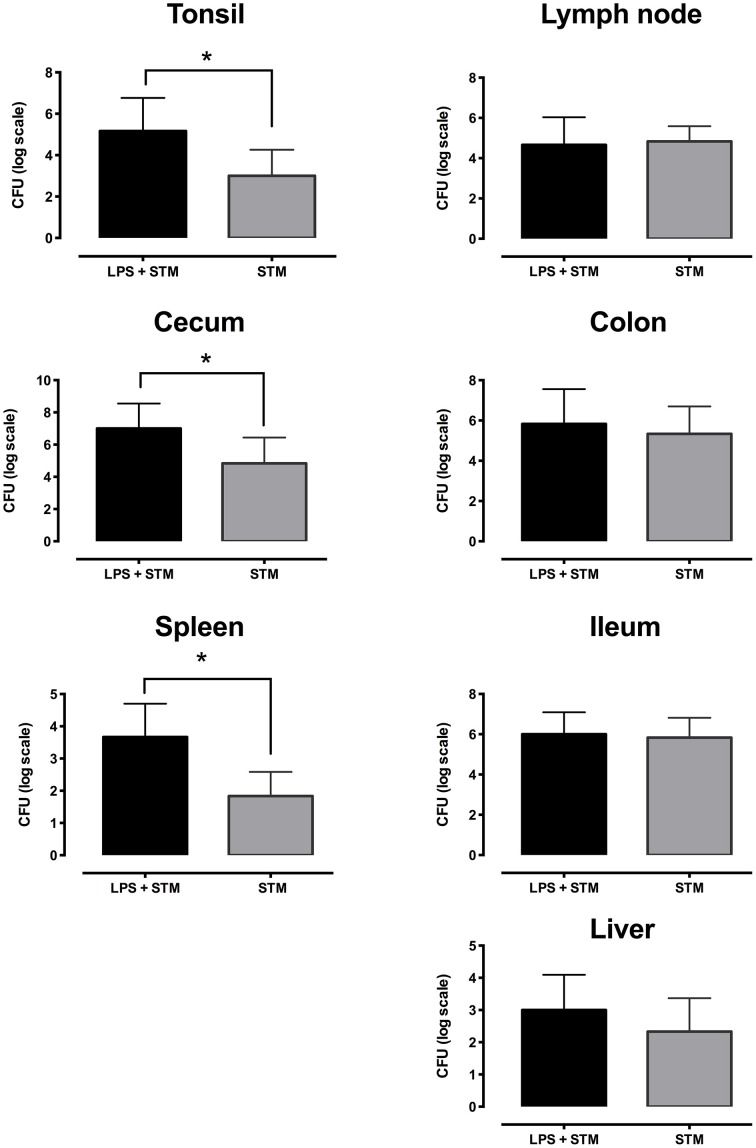
**LPS treatment raises STM14028 colonization of tonsils, cecum, and spleen of piglets**. Recovery of STM14028 from different organs at 48 h post infection of piglets treated with LPS and infected with STM14028 (LPS+STM group) or only infected with STM14028 (STM group). LPS-treatment increases the colonization of tonsils, cecum, and spleen but does not influence the colonization of mesenteric lymph nodes, colon, ileum, and liver of piglets after STM14028 infection. Data refer to one out of two separate experiments performed with comparable results. The differences between the groups were statistically significant (^*^P ≤ 0.01, Mann–Whitney unpaired *t*-test).

Piglets of the three groups were euthanized 48 h after the treatments, and STM14028 infection was assessed in different organs and tissues in order to evaluate the capability of colonizing either locally in the gut milieu, or systemically. As depicted in Figure 5, piglets treated with LPS and infected with STM14028 showed a significant increase in colonization of tonsils, cecum, and spleen, whereas in mesenteric lymph nodes, colon, ileum and liver no significant difference in STM14028 colonization was observed (Figure 5).

On the whole, these findings provide substantial evidence that LPS is able to induce an inflammatory response, which favors STM14028 survival and colonization in the intestinal and systemic compartments.

## Discussion

Intestinal inflammation, induced by both chemical treatments and infectious agents, is known to be associated with a profound dysbiosis of the colonic microbial community structure (Lupp et al., 2007). Many pathogens use inflammation and the accompanying dysbiosis for their advantage in order to overcome colonization resistance (Stecher et al., 2007). In this context, several studies based on the use of the streptomycin-treated mouse colitis model, which is characterized by absence of resident microbiota (Ahmer and Gunn, 2011), highlighted the emerging concept that inflammation of the mucosal epithelium plays a role in environmental fitness of *S*. *Typhimurium*. It has been shown, indeed, that unlike avirulent strains, wild-type *S*. *Typhimurium* is capable of out-competing commensal microbiota in re-colonization experiments after treatment with antibiotics. Furthermore, *S*. *Typhimurium* exploits inflammation to promote its own colonization, out-competing the resident microbiota (Stecher et al., 2007). This model however, despite the important contribution to the study of the pathogens-microbiota interaction in an inflammatory environment, presents several limitations. In particular, the inability of the *Salmonella* mouse model to reproduce gastroenteritis and, even more importantly, the elimination of the competing flora represent crucial differences with respect to natural *Salmonella* infection in humans. In this work, an experimental model was used based on piglets infected with a wild type strain of *S*. *Typhimurium*, STM14028, representing an ideal animal model potentially capable to overcome the intrinsic limitations of the current streptomycin-treated mouse colitis model.

It was initially observed that, after oral infection with STM14028, the immune response is rapidly activated, involving the innate compartment with a marked increase of polymorphonuclear and mono-macrophage populations in ileo-cecal lymph nodes (Figure 1). This confirms the involvement of the principal populations engaged in the response to *Salmonella* infection, already known to be relevant for the response to the LPS stimulation. Moreover, these results extend those of recent studies about the increased expression of pro-inflammatory cytokines (Knetter et al., 2015) and raised lymphocytic infiltration of the gut mucosa after *S*. *Typhimurium* oral infection of piglets (Gradassi et al., 2013).

It was investigated whether the induction of inflammation by LPS pre-treatment of mono-macrophages and porcine intestinal epithelial IPEC-J2 cells makes these cells more susceptible to STM14028 infection. The results indicate that these cells, primed with LPS, were more prone to the colonization by STM14028 when compared to the LPS-untreated control cells. Otherwise, the use of the RS-LPS antagonist, binding the TLR-4/MD-2 complex, inhibits the LPS stimulation. This significantly reduced the STM14028 intracellular colonization down to the values of LPS-untreated cells (Figure 2).

The higher colonization of STM14028 in LPS-treated cells could be considered unexpected due to the effect of LPS stimulus. It can however be hypothesized that our results are the consequence of high capability of bacterial up-take. It has been well-established, in fact, that for many facultative intracellular pathogens, as well for *Salmonella*, the key to successful infection lies in the interaction between bacteria and host macrophages. *Salmonella* is able to mount specific strategies to escape killing and survive within phagocytes (Fields et al., 1986; Groisman and Saier, 1990; Gulig et al., 1998; Ruby et al., 2012). Moreover, recent reports have revealed fascinating insights to explain how *Salmonella* exploits host response. In particular, the internalization of *Salmonella* in macrophages via TLR, able to bind *Salmonella* LPS, is a crucial factor to favor *Salmonella* virulence in that it facilitates the acidification of the phagosome, which in turn provides a protective niche for *Salmonella* (Arpaia et al., 2011). In addition, in mouse models, it has been observed that LPS present on live *Salmonella* provides an essential signal, via functional TLR-4, for macrophages to produce NO and TNF-α (Royle et al., 2003). This may be exploited by *Salmonella* to modify macrophage functions and promote growth and/or dissemination throughout the host.

Finally, the main effort was to assess whether the inflammation induced LPS-treated piglets was able to influence the colonization of STM14028. Piglets treated with LPS and infected with STM14028 showed a significant increase in body temperature (Figure 3) and the production of IL-1beta and TNF-alpha in the blood (Figure 4) at 4 h post infection. These results indicate that the onset of the LPS-mediated acute inflammation leads to cytokines production and body temperature rise, already at 4 h post infection, promoting significant increase of tonsils, cecum, and spleen STM14028-colonization, compared to the control group (Figure 5).

These results are in line with studies showing that the parenteral administration of dead Gram-negative bacteria or lipopolysaccharide exacerbated the growth of virulent *S. enterica* in mice (Hormaeche, 1990). It is also possible to envisage that the increased advantage of *Salmonella* in inflamed systemic environment can also be justified by mechanisms other than a compromised barrier integrity or dysbiosis. Moreover, Foster *et al*. demonstrated that the intravenous administration of an attenuated *Salmonella* strain can exacerbate the growth of virulent strains, which involves IL-10 production and requires TLR-4, and its signaling pathways involving the adaptor molecules, the TIR-domain-containing adapter-inducing interferon-β (TRIF), and the Myeloid differentiation primary response gene 88 (MyD88) (Foster et al., 2008). A dysregulated type I IFN response in tissues can affect fundamental regulatory circuits of innate immunity in macrophages, which turns IL-10 into a potent pro-inflammatory cytokine (Sharif et al., 2004). In addition, IL-10 and IFN-γ associated responses may cause a gain of pro-inflammatory activity, as shown in human models of endotoxemia (Lauw et al., 2000).

On the whole, these results, using piglets as model, demonstrate for the first time that in acute enteric salmonellosis, *S*. *Typhimurium* exploits the inflamed milieu to its own advantage.

## Author contributions

BC planned and performed the research, analyzed data and co-wrote the manuscript. PP designed, planned and performed the research, analyzed data and co-wrote the paper. GA designed, planned, and performed the research and analyzed data. MA planned and performed part of the research and analyzed data. RD and MP performed part of the research and contributed to the analysis of the data. ER performed cytofluorimetric analysis. JR and NM performed part of the research. LM performed Immunoenzymatic analysis. CM and LC performed *in vivo* studies. PP and CP contributed to perform technical experiments.

## Funding

This work was supported by ISS intramural research funds and by Transnational Research Project EMIDA ERA-NET “HealthyGut - Multi-focal strategies to improve gut health and reduce enteritis in poultry and pigs”, MIPAF- DM 27373/7303/2010. The research by Pesciaroli Michele was partly supported by a PICATA postdoctoral contract of the Moncloa Campus of International Excellence (UCM-UPM, Campus Moncloa, VISAVET), “HealthyGut.”

### Conflict of interest statement

The authors declare that the research was conducted in the absence of any commercial or financial relationships that could be construed as a potential conflict of interest.
